# Risk factors for multidrug-resistant tuberculosis: A worldwide systematic review and meta-analysis

**DOI:** 10.1371/journal.pone.0270003

**Published:** 2022-06-16

**Authors:** Ying Xi, Wei Zhang, Rui-Jun Qiao, Jun Tang

**Affiliations:** 1 Tuberculosis Ward 1, Shenyang Tenth People’s Hospital, Shenyang Chest Hospital, Shenyang, China; 2 Medical Department, Shenyang Tenth People’s Hospital, Shenyang Chest Hospital, Shenyang, China; The University of Georgia, UNITED STATES

## Abstract

**Background:**

Since multidrug-resistant tuberculosis (MDR-TB) is a significant public health problem worldwide, identifying associated risk factors is critical for developing appropriate control strategies.

**Methods:**

A systematic review and meta-analysis was conducted for identifying factors independently predicting MDR-TB. The random-effects model was used to determine pooled odds ratios (ORs) and respective 95% confidence intervals (CIs) for the related factors.

**Results:**

Of the 2301 retrieved reports, 28 studies were analyzed, assessing 3152 MDR-TB and 52715 DS-TB cases. Totally 22 related factors were analyzed. The pooled ORs were 1.478 (95%CI 1.077–2.028) for positive sputum AFB smear, 1.716 (95%CI 1.149–2.564) for lung cavity, 6.078 (95%CI 2.903–12.725) for previous TB disease and 5.427 (95%CI 3.469–8.490) for a history of anti-TB therapy. All Z test p values were below 0.05, indicating these parameters were significantly associated with MDR-TB.

**Conclusions:**

Positive sputum AFB smear, lung cavity, previously diagnosed TB and a history of anti-TB therapy are significant risk factors for MDR-TB, which are independent of the clinical setting worldwide. Increased attention should be paid to cases with such parameters to achieve more effective TB control and avoid MDR-TB through the development of a global policy.

## Introduction

Tuberculosis (TB) is a chronic infectious disease caused by *Mycobacterium tuberculosis* (MTB). TB constitutes one of the top 10 death causes globally and the leading cause of death from a single infectious agent. As with other antibiotics, antibiotic resistance is inevitable with anti-TB agents. Drug-resistant TB (DR-TB) is the main challenge of the WHO Global TB Programme due to its high risk of relapse, treatment failure, prolonged transmission of the bacilli, and death [1]. Multidrug-resistant tuberculosis (MDR-TB) is defined as TB caused by MTB strains with resistance to at least both rifampicin and isoniazid. Increasing MDR-TB incidence is one of the many factors explaining the resurgence of the global TB epidemic. According to the global Tuberculosis Report 2020 [2], approximately 10.0 million individuals had TB diagnosis in 2019 globally, including 206,030 with MDR/RR-TB (rifampicin-resistant tuberculosis, RR-TB), representing a 10% increase from 186,883 in 2018. According to global data in 2019, 61% of people with bacteriologically confirmed TB were tested positive for rifampicin resistance, up from 51% in 2017 and 7% in 2012. In accordance with WHO guidelines, detection of MDR/RR-TB requires bacteriological confirmation of TB and testing for drug resistance using rapid molecular tests, culture methods or sequencing technologies.

MDR-TB often occurs in individuals with failed repeated irregular treatment with standard short-course and relapse regimens, representing a source of relapse and refractory treatment. Patients with MDR-TB are practically incurable by standard first-line TB drugs [3, 4]. Therefore, MDR-TB emergence remains a threat to human health, especially in some high-burden countries [5]. Poorer treatment outcome, prolonged treatment (approximately two years), elevated treatment cost, and multiple complications make MDR-TB a disease with higher complexity compared with sensitive TB [6]. MDR-TB has a high mortality rate and poses a great threat to healthy individuals. Due to the spread of MDR-TB, TB may once again become an "incurable disease".

The occurrence of MDR-TB possibly involves many different factors such as race, socio-economic situation, culture, lifestyle, epidemiology, medical condition and detection techniques. Therefore, identifying risk factors for MDR-TB is critical. Multiple investigations examining risk factors for MDR-TB have been carried out in several countries [7–9]. However, some of the reported results were inconsistent or conflicting. In addition, the findings of these studies were limited due to the differences in geographical location. Therefore, a worldwide systematic review and meta-analysis was carried out for reasonably identifying factors independently predicting MDR-TB versus drug-susceptible TB, which were independent of the setting.

## Materials and methods

This work was reported according to the Preferred Reporting Items for Systematic Reviews and Meta-Analyses (PRISMA) Statement.

### Search strategy

We searched for relevant studies published in three databases, including PubMed, EmBase and the Cochrane Library. All publications were queried till March 23, 2021, by two independent investigators (YX and WZ). We included observational predictive studies without language restriction, including case-control, cohort and cross-sectional studies.

The complete search strategy used for PubMed was: (("Tuberculosis, Multidrug-Resistant" [Mesh]) OR ((((((((((Multidrug-Resistant Tuberculosis[Title/Abstract]) OR (Tuberculosis, Multidrug Resistant[Title/Abstract])) OR (Tuberculosis, MDR[Title/Abstract])) OR (MDR Tuberculosis[Title/Abstract])) OR (Tuberculosis, Multi-Drug Resistant[Title/Abstract])) OR (Multi-Drug Resistant Tuberculosis [Title/Abstract])) OR (Tuberculosis, Multi Drug Resistant[Title/Abstract])) OR (Tuberculosis, Drug-Resistant[Title/Abstract])) OR (Drug-Resistant Tuberculosis[Title/Abstract])) OR (Tuberculosis, Drug Resistant[Title/Abstract]))) AND (("Risk Factors"[Mesh]) OR ((((((((((((Factor, Risk[Title/Abstract]) OR (Risk Factor[Title/Abstract])) OR (Health Correlates[Title/Abstract])) OR (Correlates, Health[Title/Abstract])) OR (Risk Scores[Title/Abstract])) OR (Risk Score[Title/Abstract])) OR (Score, Risk[Title/Abstract])) OR (Risk Factor Scores[Title/Abstract])) OR (Risk Factor Score[Title/Abstract])) OR (Score, Risk Factor[Title/Abstract])) OR (Population at Risk[Title/Abstract])) OR (Populations at Risk[Title/Abstract]))).

The complete search strategy used for EmBase was: ’’multidrug resistant tuberculosis’/exp OR ’tuberculosis, multidrug-resistant’:ab,ti OR ’tuberculosis, multidrug resistant’:ab,ti OR ’tuberculosis, mdr’:ab,ti OR ’mdr tuberculosis’:ab,ti OR ’tuberculosis, multi-drug resistant’:ab,ti OR ’multi-drug resistant tuberculosis’:ab,ti OR ’tuberculosis, multi drug resistant’:ab,ti OR ’tuberculosis, drug-resistant’:ab,ti OR ’drug-resistant tuberculosis’:ab,ti OR ’tuberculosis, drug resistant’:ab,ti’ AND ’’risk factor’/exp OR ’risk factors’:ab,ti OR ’factor, risk’:ab,ti OR ’health correlates’:ab,ti OR ’correlates, health’:ab,ti OR ’risk scores’:ab,ti OR ’risk score’:ab,ti OR ’score, risk’:ab,ti OR ’risk factor scores’:ab,ti OR ’risk factor score’:ab,ti OR ’score, risk factor’:ab,ti OR ’population at risk’:ab,ti OR ’populations at risk’:ab,ti’.

The complete search strategy used for Cochrane Library was: (((multidrug resistant tuberculosis):ti,ab,kw OR (tuberculosis, multidrug-resistant):ti,ab,kw OR (tuberculosis, multidrug resistant):ti,ab,kw OR (tuberculosis, mdr):ti,ab,kw OR (mdr tuberculosis):ti,ab,kw) OR ((tuberculosis, multi-drug resistant):ti,ab,kw OR (multi-drug resistant tuberculosis):ti,ab,kw OR (tuberculosis, multi drug resistant):ti,ab,kw OR (tuberculosis, drug-resistant):ti,ab,kw OR (drug-resistant tuberculosis):ti,ab,kw) OR ((tuberculosis, drug resistant):ti,ab,kw)) AND (((risk factor):ti,ab,kw OR (risk factors):ti,ab,kw OR (factor, risk):ti,ab,kw OR (health correlates):ti,ab,kw OR (correlates, health):ti,ab,kw) OR ((risk scores):ti,ab,kw OR (risk score):ti,ab,kw OR (score, risk):ti,ab,kw OR (risk factor scores):ti,ab,kw OR (risk factor score):ti,ab,kw) OR ((score, risk factor):ti,ab,kw OR (population at risk):ti,ab,kw OR (populations at risk):ti,ab,kw)).

### Study eligibility criteria

The following selection criteria were utilized for eligibility: (1) TB-patients, 15 years of age or older; (2) MDR-TB and drug-susceptible tuberculosis (DS-TB) defined by microbiological verification (i.e., DST or rapid molecular tests to detect DNA and mutations associated with drug resistance); (3) observational trials providing data.

In case-control and cross-sectional trials, MDR-TB patients with MTB strains with resistance to at least both rifampicin (RMP) and isoniazid (INH) were examined, and individuals with DS-TB and sputum culture demonstrating MTB sensitivity to first-line anti-TB agents, i.e., INH, RMP, ethambutol (EMB) and streptomycin (SM), as controls. For cohort studies, DS-TB cases were included as individuals at risk, with MDR-TB considered the outcome.

For eligible reports involving the same research group, the most recent publication with the highest patient number was considered. If including different parameters, the relevant articles were all included to consider all factors, but participants were not counted repeatedly.

Reports only examining certain high-risk MDR-TB populations, including TB cases with diabetes mellitus, HIV or cancer, were excluded.

### Data extraction

Two independent investigators (YX and WZ) examined all titles and abstracts independently, retrieving reports meeting the eligibility criteria for further full-text assessment. Any disagreement was resolved by another investigator (RJQ). The level of disagreement was determined as reported previously based on Cohen’s Kappa [10].

Data extraction involved first author, publication year, study country, study period, sample size (cases and controls), associated risk factors and study quality.

### Quality assessment

Study quality was examined by two investigators in an independent manner based on the Newcastle-Ottawa Quality Assessment Scale (NOS) (McPheeters, 2012), considering selection, comparability and outcome. Studies were given a quality score, and those with NOS score ≤5 were considered low-quality trials and excluded from the current meta-analysis.

### Statistical analysis

For data analysis, Stata 15.1 and Review Manager version 5.4 were utilized. Dichotomous variables were applied to various factors analyzed. To determine the associations of different parameters with MDR-TB, risk factors with similar definitions were pooled utilizing Mantel-Haenszel ORs and 95%CIs in a random-effects model. *P*<0.05 indicated statistical significance.

Study heterogeneity was determined by the Cochran *Q* and *I*^2^ statistic tests. A *p*-value of heterogeneity below 0.05 was defined as considerable heterogeneity. The *I*^2^ test was performed to assess the magnitude of heterogeneity among trials, and *I*^2^>50% indicated moderate-to-high heterogeneity [11]. Sensitivity was examined for risk factors with heterogeneous data, excluding each study in turn, to determine the effect sizes of various risk factors.

Funnel plots were generated to examine publication bias, and analyzed by the Begg’s and Egger’s tests. *P*<0.05 was the cutoff for significance in publication bias.

## Results

**Fig 1:**
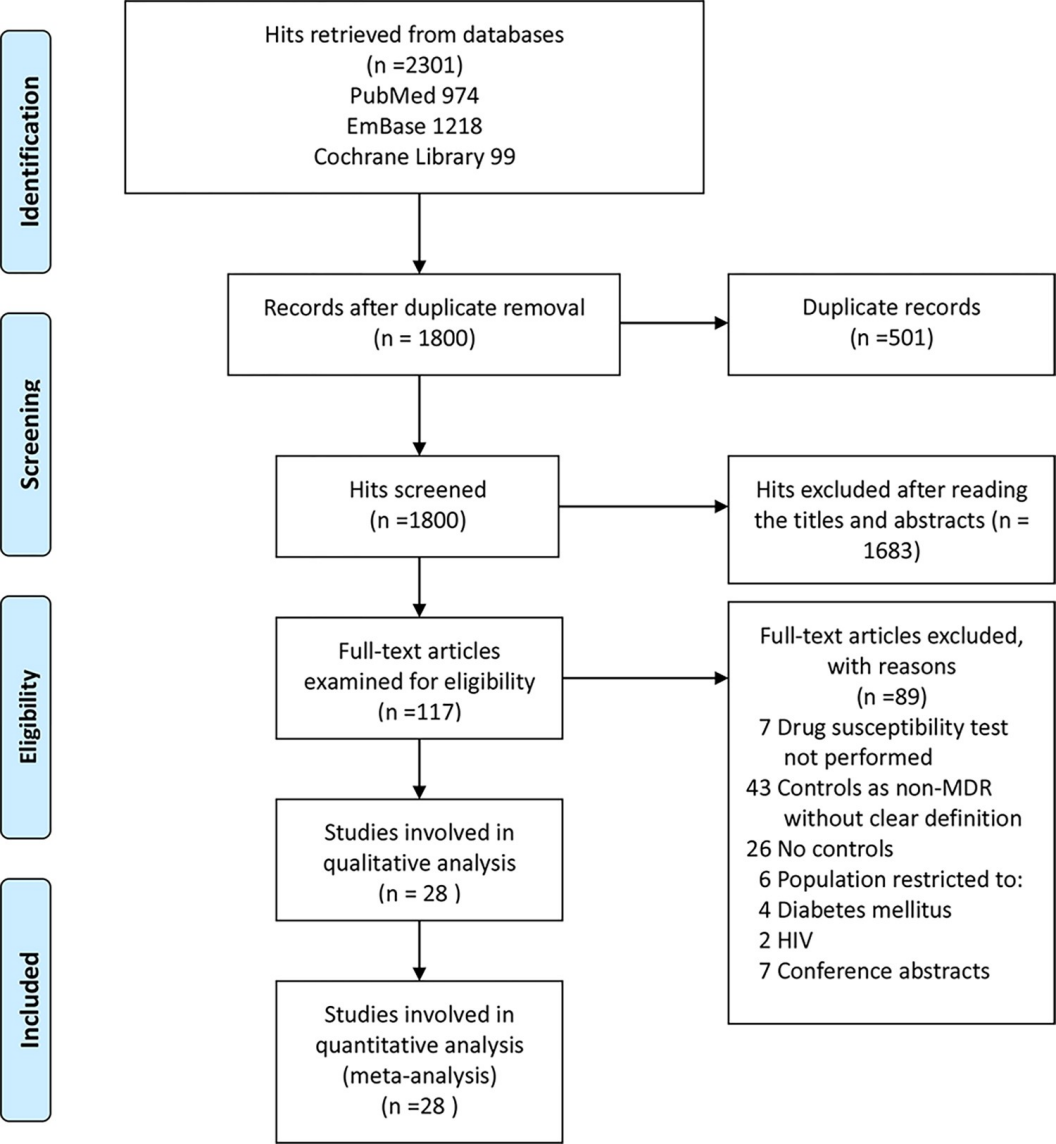
Study flowchart.

A total of 2301 original publications were initially found in the PubMed, EmBase and Cochrane Library databases. Of these, 501 duplications were removed, and 1683 reports were ruled out by examining their titles and abstracts. Finally, 28 studies were meta-analyzed, with 3152 MDR-TB cases and 52715 DS-TB controls. Fig 1 shows the study flowchart. In the title and abstract screening process, 54 discrepancies occurred between the two primary investigators, with 97% agreement (good) and a reliability coefficient of 0.796 (good) according to Cohen’s Kappa. In full-text screening, the investigators disagreed about 7 of the 117 examined studies, indicating good agreement (94%) and reliability (0.848) according to Cohen’s kappa. The general information and quality assessment scores of all meta-analyzed reports are summarized in Table 1.

**Table 1:** General information and NOS scores of meta-analyzed studies.

Author and Year (Ref.)	Country	Study design	Study period	Number of cases (MDR-TB)	Number of controls (DS-TB)	Risk factors identified	NOS Marks
Shin et al. 2020 [12]	Korea	Case-control	2010–2016	90	90	01,10,13,15,16	7
Glasauer et al. 2019 [13]	Germany	Cross-sectional	2008–2017	556	22904	01,02,16,19	6
Stosic et al. 2018 [14]	Serbia	Case-control	2009–2014	31	93	01,03,05,08,09,1012,20,22	7
Gaborit et al. 2018 [15]	France	Case-control	2002–2013	44	90	01,07,08,09,10,11,18,19	7
Gao et al. 2016 [16]	China	Cohort	2008–2010	17	1609	01,02,20	8
Li et al. 2016 [17]	China	Case-control	2007–2012	325	613	01,14,15,20,21	7
Yin et al. 2016 [18]	China	Cross-sectional	2006 and 2012	183	411	01,21	6
Wang et al. 2016 [19]	China	Cross-sectional	2004–2005	160	1867	14	6
Chuchottaworn et al. 2015 [20]	Thailand	Case-control	2007–2013	145	145	01,02,04,10,11,15,22	8
Elmi et al. 2015 [21]	Malaysia	Case-control	2010–2014	105	209	01,07,08,09,10,11,12,15,18,20,22	8
Mor et al. 2014 [22]	Israel	Case-control	1999–2010	207	3107	01,07,11,13,16,20	8
Li et al. 2014 [23]	China	Case-control	2011	110	110	01,04,05,06,08,09,17,18	7
Zhao et al. 2012 [24]	China	Cross-sectional	2004–2005	401	2426	01,02,06,20	7
Coelho et al. 2012 [25]	Brazil	Cross-sectional	2000–2004	32	157	08,10,11,15,19,21	6
Ayaz et al. 2012 [26]	Pakistan	Cross-sectional	2006–2009	43	770	01,05,17,18	7
He et al. 2011 [27]	China	Case-control	2007–2009	100	97	01,06,14,15,18,20	7
Fox et al. 2011 [28]	Israel	Case-control	2002–2009	44	508	01,08,09,10,11,15,20	8
Massi et al. 2011 [29]	Indonesia	Cross-sectional	2008	16	120	01,02,04,08,09,10,15,18,20	6
Balaji et al. 2010 [30]	India	Case-control	2002–2007	30	117	01,11,15,16	6
Diande et al. 2009 [31]	Burkina Faso	Case-control	2005–2006	56	304	01,02,06,08,11,18,20	7
Shen et al. 2009 [32]	China	Case-control	2000–2006	333	7018	01,03,10,13,15,20	8
O’Riordan et al. 2008 [33]	England	Case-control	1982–2004	42	84	01,13,19	7
Tanrikulu et al. 2008 [34]	Turkey	Cross-sectional	2001–2005	13	84	01,03,10,20	6
De Souza et al. 2006 [35]	Brazil	Case-control	2000–2004	12	36	01,08,10,13,18,20,21	8
El Sahly et al. 2006 [36]	The United States	Case-control	1995–2001	15	1977	01,05,07,08,09,11,18,19	8
Conaty et al. 2004 [37]	England and Wales	Case-control	1993–1994 and 1998–2000	140	8700	01,02,11,13,16	7
Schaberg et al. 1995 [38]	Germany	Case-control	1987–1993	39	913	01,08,13,15,20	7
Pearson et al. 1992 [39]	The United States	Case-control	1989–1991	23	23	01,11,16	7

*Notes*: 01, Sex; 02, Age group, years; 03, Residential area; 04, Education; 05, Marital status; 06, Occupation; 07, History of incarceration; 08, Alcohol abuse; 09, Smoking; 10, Diabetes mellitus; 11, HIV infection; 12, COPD; 13, Sputum AFB smear; 14, Beijing genotype; 15, Cavity visible on radiograph; 16, Site of TB infection; 17, BCG scar; 18, Close contact with a TB patient; 19, Past TB history; 20, Previous history of TB treatment; 21, Treatment abandonment; 22, Known outcome of previous TB treatment.

Because a study [14] was performed using representative MTB strains isolated from another study [19], and these two reports assessed different factors, they were both included but the number of participants was not included repeatedly.

Totally 22 related factors were meta-analyzed. These factors were categorized as demographic characteristics (i.e., sex, age group, residential area, education, marital status, occupation, and a history of incarceration), bad habits (i.e., alcohol abuse and smoking), comorbidities (i.e., diabetes mellitus, HIV infection and COPD) and TB-related clinical characteristics (i.e., sputum AFB smear, Beijing genotype, lung cavity visible on radiograph, site of TB infection, presence of a BCG scar, close contact with an individual with TB, a history of TB disease, a history of anti-TB treatment, directly observed treatment [DOT] and known outcome of previous anti-TB treatment). Statistical results for all these factors are shown in Table 2.

**Table 2:** Pooled risk estimates for various factors associated with MDR-TB.

Risk factors	Study numbers	Effect estimate		Heterogeneity		Publication bias	
		OR (95%CI)	*p*	I^2^	*p*	Begg’s *p* value	Egger’s *p* value
**Demographic features**							
Sex (Male/Female)	26 [12–18, 20–24, 26–39]	1.015(0.872–1.180)	0.850	55.2%	0.000	0.597	0.710
Age group (<40 years/≥40 years)	7 [16, 20, 24, 29, 31, 37]	1.131(0.685–1.870)	0.630	90.8%	0.000	0.764	0.163
Residence (Rural and Suburban/Urban area)	3 [14, 32, 34]	0.863(0.366–2.032)	0.736	75.7%	0.016	1.000	0.526
Education (Secondary or above/Illiteracy or primary)	3 [20, 23, 29]	1.061(0.750–1.503)	0.737	0.0%	0.821	1.000	0.168
Marital status, Married (Yes/No)	4 [14, 23, 26, 36]	0.878(0.382–2.018)	0.759	79.0%	0.003	0.308	0.516
Occupation (Farmer/Other)	4 [23, 24, 27, 31]	0.793(0.485–1.297)	0.356	67.4%	0.027	0.308	0.691
History of incarceration (Yes/No)	4 [15, 21, 22, 36]	1.443(0.299–6.969)	0.648	79.0%	0.003	1.000	0.929
**Bad behaviors**							
Alcohol abuse (Yes/No)	11 [14, 15, 21, 23, 25, 28, 29, 31, 35, 36, 38]	0.954(0.629–1.447)	0.825	57.9%	0.008	0.436	0.241
Smoking (Yes/No)	7 [14, 15, 21, 23, 28, 29, 36]	0.779(0.542–1.119)	0.177	36.4%	0.150	0.548	0.862
**Comorbidities**							
Diabetes mellitus (Yes/No)	11 [12, 14, 15, 30, 21, 25, 28, 29, 32, 34, 35]	1.117(0.841–1.483)	0.444	16.8%	0.284	0.276	0.231
HIV infection (Negative/Positive)	11 [15, 20, 21, 22, 25, 28, 30, 31, 36, 37, 39]	0.813(0.388–1.073)	0.583	85.0%	0.000	0.876	0.565
COPD (No/Yes)	2 [14, 21]	1.186(0.066–21.260)	0.908	91.4%	0.001	1.000	NA
**TB-related parameters**							
Sputum AFB smear (Positive/Negative)	7 [12, 22, 32, 33, 35, 37, 38]	1.478(1.077–2.028)	0.016	61.9%	0.015	0.548	0.749
Beijing genotype (Yes/No)	3 [17, 19, 27]	2.221(0.608–8.117)	0.227	86.4%	0.001	1.000	0.124
Lung cavity visible on radiograph (Yes/No)	11 [12, 17, 20, 21, 25, 27–30, 32, 38]	1.716(1.149–2.564)	0.008	83.4%	0.000	1.000	0.327
Site of TB infection (Pulmonary/Extra-pulmonary)	6 [12, 13, 22, 30, 37, 39]	1.232(0.834–1.820)	0.295	52.8%	0.006	0.133	0.012
BCG scar (Present/Absent)	2 [23, 26]	1.123(0.698–1.807)	0.632	25.6%	0.246	1.000	NA
Close contact with a TB patient (No/Yes)	9 [15, 21, 23, 26, 27, 29, 31, 35, 36]	0.743(0.446–1.237)	0.253	68.9%	0.001	0.348	0.343
History of TB disease (Yes/No)	5 [13, 15, 25, 33, 36]	6.078(2.903–12.725)	0.000	79.3%	0.001	0.462	0.712
History of anti-TB therapy (Yes/No)	16 [14, 16–18, 21, 22, 24, 25, 27–29, 31, 32, 34, 35, 38]	5.427(3.469–8.490)	0.000	89.3%	0.000	0.444	0.531
DOT (Yes/No)	3 [17, 25, 35]	0.229(0.049–1.070)	0.061	86.8%	0.001	1.000	0.818
Known outcome of previous TB therapy (Cure or complete/Failure or default)	3 [14, 20, 21]	0.477(0.206–1.102)	0.083	64.4%	0.006	1.000	0.094

*Notes*: HIV, human immunodeficiency virus; COPD, Chronic Obstructive Pulmonary Disease; BCG, Bacillus Calmette Guerin; DOT, directly observed treatment; NA, not available.

**Fig 2:**
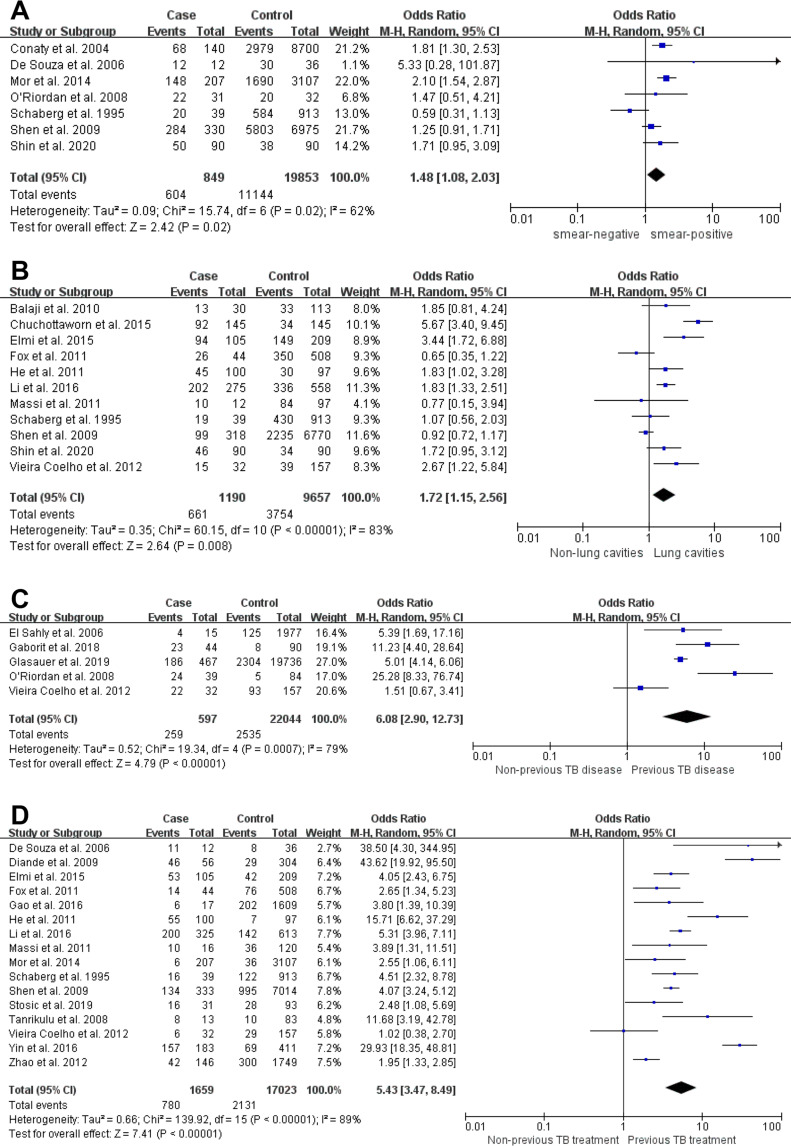
Forest plots showing associations of sputum AFB smear. (A), lung cavity visible on radiograph (B), previous TB disease (C) and previous anti-TB treatment (D) with multidrug-resistant TB.

Sputum AFB smear positivity (OR = 1.478, 95%CI 1.077–2.028), lung cavity (OR = 1.716, 95%CI 1.149–2.564), a history of TB disease (OR = 6.078, 95%CI 2.903–12.725) and a history of anti-TB treatment (OR = 5.427, 95%CI 3.469–8.490) had pooled ORs greater than 1, with *Z* test p<0.05, indicating they were significant risk factors for MDR-TB. Forest plots are depicted in Fig 2. The pooled ORs of the remaining factors (i.e., sex, age, residential area, education, marital status, occupation, a history of incarceration, alcohol abuse, smoking, diabetes mellitus, HIV infection, COPD, Beijing genotype, site of TB infection, presence of a BCG scar, close contact with an individual with TB, DOT and known outcome of previous anti-TB treatment) included 1 and the associated *p-*values were > 0.05, indicating these parameters could not independently predict MDR-TB.

Heterogeneity analysis revealed *I*^2^ values for education, smoking, diabetes mellitus and presence of a BCG scar were < 50%, with chi-square test p>0.05, indicating no heterogeneity among the included studies. However, *I*^2^ values for the remaining factors (i.e., sex, age, residential area, marital status, occupation, a history of incarceration, alcohol abuse, HIV infection, COPD, sputum AFB smear, Beijing genotype, lung cavity, site of TB infection, close contact with an individual with TB, a history of TB disease, previous anti-TB therapy, DOT and known outcome of previously administered anti-TB therapy) were > 50%, with chi-square test p<0.05, suggesting heterogeneity among the examined studies. Therefore, we carried out a sensitivity analysis for identifying the source of heterogeneity through removal of every study in turn. The results showed no reported trial significantly affected the pooled effects.

The funnel plots showed no asymmetry. In addition, Begg’s and Egger’s *p*-values were > 0.05, indicating no publication bias.

## Discussion

This work examined risk factors for MDR-TB based on demographics, bad living habits, complications and TB-related features. We found sputum AFB smear positivity, lung cavity, a history of TB and a history of anti-TB therapy were significant risk factors for MDR-TB, independent of the setting. On the contrary, sociodemographic characteristics (i.e., sex, age group, residential area, education, marital status, occupation, and a history of incarceration), bad habits (i.e., alcohol abuse and smoking), comorbidities (i.e., diabetes mellitus, HIV infection and COPD), and other TB-related characteristics (i.e., Beijing genotype, site of TB infection, a history of BCG scar, close contact with an individual with TB, DOT and known outcome of previous anti-TB treatment) showed no overt associations with MDR-TB.

TB represents an extremely dangerous human disease, which is considered the greatest killer among all diseases; it has probably been present throughout the world for thousands of years. The emergence and spread of drug-resistant TB mostly involve interdependent parameters related to patient features, the health care system [40] and MTB’s response allowing strains with elevated severity, reduced treatability and decreased detectability under selection pressure [41]. MDR-TB is a threat to human health because of challenging and expensive diagnosis and treatment. Although slightly less drug-susceptible TB cases are currently reported worldwide, MDR-TB diagnoses are on the rise, indicating the need for progress in this field. To ensure patients adhere to the full therapeutic course, helping them take the tablets regularly and having a health worker meet with them and observe the drugs being swallowed has been implemented. Failure to do this promotes acquired drug resistance. The serious epidemic of MDR-TB is considered an important public health issue in many nations, and constitutes a huge factor hampering effective TB control worldwide, which highlights the usefulness of universal drug susceptibility testing. Nowadays, drug resistance can be diagnosed either by phenotypic (culture) or genotypic (DNA) assays, which are much faster and can be done in one day or less.

Similarly, identifying risk factors for MDR-TB is important. There are many differences among studies assessing risk factors for MDR-TB because of region and sample size differences, scattered factors and inconsistent results, among others. Our analysis systematically evaluated global risk factors for MDR-TB independent of the geographical setting, which could help develop an effective prevention and control strategy, to achieve the purpose of cause prevention (primary prevention).

As shown above, sputum AFB smear positivity (OR = 1.478, 95%CI 1.077–2.028) independently predicted MDR-TB, corroborating a previous study [42]. Smear-positive PTB represents the disease form with highest infectivity and transmissibility among humans, and can be prevented by airborne precautions [43]. This smear-positivity is associated with infectiousness likely because smear-positive cases expel larger amounts of MTB into the environment compared with smear-negative cases, as MTB organisms are only observed by sputum microscopy at sufficiently high amounts [44]. Sputum AFB smear-positivity indicates an elevated bacterial burden. In addition, a high bacterial load may reflect an elevated microbial burden, whose treatment would be more challenging. Smear positivity helps both TB and MDR-TB spread, increasing the probability of drug resistance. Isoniazid and rifampicin both exhibit spontaneous resistance mutations. Mutations in specific genes are associated with drug-resistance. MTB acquires resistance to antimicrobial drugs through the selection of bacteria with mutations in resistance genes [45]. Previous studies have shown that antibiotic resistance-related mutations can impair bacterial fitness [46, 47], and the acquisition of compensatory mutations in drug-resistant strains can restore their ability to survive. A molecular study suggested that drug-resistant strains of MTB may be as transmissible as pan-sensitive strains [48]. So, it is important to appropriately treat smear-positive patients to achieve a successful control of TB. Furthermore, MDR-TB cases have markedly increased sputum smear-positivity rate compared with drug-sensitive TB cases (MDR-TB in 80.0% vs drug-sensitive TB in 53.3%) [49].

Also, this study showed that MDR-TB occurrence is elevated in individuals with lung cavities in comparison with those with no lung cavities (OR = 1.716, 95%CI 1.149–2.564), which was consistent with a previous study [7]. TB caseous necrosis can easily form lung cavities if not timely treated. Cavitary tuberculosis is caused by lung injury, and reflects macroscopic openings developed between the infection site and the airways that promote bacterial expectoration [50]. These cavities may remain for a long time in case of inappropriate treatment; cavity wall thickening might occur, as well as fibrotic lesions, which may progress to chronic fiber cavity pulmonary TB [8]. Drugs administered orally or intravenously rarely achieve effective circulating concentrations in cavities, promoting MDR occurrence [51]. This constitutes a major barrier to TB control. Investigators have injected drugs via percutaneous lung biopsy into cavities to treat pulmonary TB cavities [52]. Therefore, three levels of prevention should be implemented to prevent the occurrence of cavitary tuberculosis, including early detection, timely treatment, and the implementation of interventional treatment for the associated lesions [53].

Furthermore, the above results were consistent with a previous meta-analysis [42] showing that previously diagnosed TB and anti-TB therapy constitute the most consistently significant risk factors for MDR-TB, not relying on the setting. These findings also corroborate another report [54]. Our analysis showed the odds of having MDR-TB was 6.078 times higher in individuals with previous TB disease compared with those with no history of TB disease; meanwhile, individuals with a history of anti-TB therapy were 5.427 times more prone to develop MDR-TB compared with cases with no previous TB therapy. Our results confirm a tight association of previous anti-TB with MDR-treatment TB infection [55]. Prior exposure to anti-TB agents in amounts insufficient for complete treatment is well-known as a trigger of drug resistance, according to global surveillance data [56, 57]. Prior exposure to anti-TB agents may significantly increase the odds of the infecting strains acquiring multidrug resistance, especially in case of non-compliance. Most MDR-TB cases are due to poor adherence to TB medications, irregular use of drugs, interrupted drug supplies, physician error, and accessibility of drugs without a prescription for adequate treatment [58]. Consequently, TB therapy should be standardized, and closely monitoring of TB cases is indispensable. DOT is the most effective method for ensuring proper medication utilization around the world [59]. At the same time, implementing the DOT strategy remains critical for effectively controlling drug-resistant TB and MDR-TB, particularly supervising TB cases during therapy. To retreat patients, DST is required when starting anti-TB therapy, and the respective drugs would be selected. So rapid DST techniques are meaningful, especially in patients with high risk of drug-resistant TB, to rapidly commence adequate treatment before phenotypic DST results become available. Moreover, other TB management and control measures must also be improved, i.e., clinical guideline update, continuous training of health professionals, and supervision and support of healthcare workers during service deliveries.

The limitations of the present meta-analysis should be mentioned. Firstly, no prospective randomized controlled trials were included, indicating potential risk of bias (selection and information biases), which results from the observational nature of the analyzed trials. Secondly, not all related factors in the included reports could be analyzed due to an insufficient number of trials and the availability of data to permit meaningful meta-analysis, including COPD, Beijing genotype, BCG scar, DOT and known outcome of previous TB therapy. However, Beijing M. tb strains are more likely to be MDR-TB than non-Beijing M. tb ones, according to previous studies [60–62]. Thirdly, obvious heterogeneity was found for several analyses, e.g., based on age, HIV infection, COPD, Beijing genotype, lung cavity, a history of TB treatment and DOT. However, based on sensitivity analysis, we could not determine the source of heterogeneity. Such heterogeneity might result from distinct case definitions, study designs and demographic features, among others. Hence, further in-depth studies, i.e., larger, prospective randomized controlled trials, should be conducted to verify these results. In addition, further in-depth regional studies are necessary to assess the associations of sociodemographic characteristics with MDR-TB.

## Conclusions

Sputum AFB smear positivity, lung cavity, previously diagnosed TB and previous anti-TB therapy are significant risk factors for MDR-TB, not depending on the setting worldwide. Considering these findings, increasing attention should be paid to these patients for a more efficient TB control to prevent MDR-TB. Continuous MDR-TB spread represents an urgent and tough challenge for controlling TB globally [3]. Early detecting MDR-TB is a critical part of TB control programs. Revealing risk factors for MDR-TB is essential and necessary in designing a global policy for public health interventions. Improving the education of individuals with TB and expanding routine DST should be implemented.

## Supporting information

S1 Checklist PRISMA checklist for systematic review and meta-analysis. (PDF)

S1 File Search terms and the syntax from searched databases. (DOCX)

S2 File Newcastle-Ottawa quality assessment scale used in the systematic review. (DOCX)

## Abbreviations

TB: Tuberculosis
MTB: *Mycobacterium tuberculosis*
DR-TB: drug-resistant TB
MDR-TB: multidrug-resistant tuberculosis
RR-TB: rifampicin-resistant tuberculosis
PRISMA: the Preferred Reporting Items for Systematic Reviews and Meta-Analyses
DS-TB: drug-susceptible tuberculosis
DST: drug susceptibility testing
OR: odds ratio
CI: confidence interval
RMP: rifampicin
INH: isoniazid
EMB: ethambutol
SM: streptomycin
NOS: the Newcastle-Ottawa Quality Assessment Scale
HIV: human immunodeficiency virus
COPD: chronic obstructive pulmonary disease
AFB: acid-fast-bacilli
BCG: Bacillus Calmette Guerin vaccine
DOT: directly observed treatment
DNA: deoxyribonucleic acid
PTB: pulmonary tuberculosis
