# Supplementary material for: Risk factors for multidrug-resistant tuberculosis: A worldwide systematic review and meta-analysis
Source: PLoS One. 2022 Jun 16;17(6):e0270003. doi: 10.1371/journal.pone.0270003 (PMC9202901; doi:10.1371/journal.pone.0270003)
Supplement: S1 File — (DOCX) [file pone.0270003.s002.docx]

**S1 File. Search terms and the syntax from searched databases**

**PubMed**

1. “Tuberculosis, Multidrug-Resistant”

2. “Multidrug-Resistant Tuberculosis”

3. “Tuberculosis, Multidrug Resistant”

4. “Tuberculosis, MDR”

5. “MDR Tuberculosis”

6. “Tuberculosis, Multi-Drug Resistant”

7. “Multi-Drug Resistant Tuberculosis”

8. “Tuberculosis, Multi Drug Resistant”

9. “Tuberculosis, Drug-Resistant”

10. “Drug-Resistant Tuberculosis”

11. “Tuberculosis, Drug Resistant”

12. “Risk Factors”

13. “Factor, Risk”

14. “Risk Factor”

15. “Health Correlates”

16. “Correlates, Health”

17. “Risk Scores”

18. “Risk Score”

19. “Score, Risk”

20. “Risk Factor Scores”

21. “Risk Factor Score”

22. “Score, Risk Factor”

23. “Population at Risk”

24. “Populations at Risk”

Search strings (all inclusive):

(1 OR 2 OR 3 OR 4 OR 5 OR 6 OR 7 OR 8 OR 9 OR 10 OR 11) AND (12 OR 13 OR 14 OR 15 OR 16 OR 17 OR 18 OR 19 OR 20 OR 21 OR 22 OR 23 OR 24)

**EmBase**

1. “multidrug resistant tuberculosis”

2. “tuberculosis, multidrug-resistant”

3. “tuberculosis, multidrug resistant”

4. “tuberculosis, mdr”

5. “mdr tuberculosis”

6. “tuberculosis, multi-drug resistant”

7. “multi-drug resistant tuberculosis”

8. “tuberculosis, multi drug resistant”

9. “tuberculosis, drug-resistant”

10. “drug-resistant tuberculosis”

11. “tuberculosis, drug resistant”

12. “risk factor”

13. “risk factors”

14. “factor, risk”

15. “health correlates”

16. “correlates, health”

17. “risk scores”

18. “risk score”

19. “score, risk”

20. “risk factor scores”

21. “risk factor score”

22. “score, risk factor”

23. “population at risk”

24. “populations at risk”

Search strings (all inclusive):

(1 OR 2 OR 3 OR 4 OR 5 OR 6 OR 7 OR 8 OR 9 OR 10 OR 11) AND (12 OR 13 OR 14 OR 15 OR 16 OR 17 OR 18 OR 19 OR 20 OR 21 OR 22 OR 23 OR 24)

**Cochrane Library**

1. “multidrug resistant tuberculosis”

2. “tuberculosis, multidrug-resistant”

3. “tuberculosis, multidrug resistant”

4. “tuberculosis, mdr”

5. “mdr tuberculosis”

6. “tuberculosis, multi-drug resistant”

7. “multi-drug resistant tuberculosis”

8. “tuberculosis, multi drug resistant”

9. “tuberculosis, drug-resistant”

10. “drug-resistant tuberculosis”

11. “tuberculosis, drug resistant”

12. “risk factor”

13. “risk factors”

14. “factor, risk”

15. “health correlates”

16. “correlates, health”

17. “risk scores”

18. “risk score”

19. “score, risk”

20. “risk factor scores”

21. “risk factor score”

22. “score, risk factor”

23. “population at risk”

24. “populations at risk”

Search strings (all inclusive):

(1 OR 2 OR 3 OR 4 OR 5 OR 6 OR 7 OR 8 OR 9 OR 10 OR 11) AND (12 OR 13 OR 14 OR 15 OR 16 OR 17 OR 18 OR 19 OR 20 OR 21 OR 22 OR 23 OR 24)
