# Supplementary material for: Risk factors for multidrug-resistant tuberculosis: A worldwide systematic review and meta-analysis
Source: PLoS One. 2022 Jun 16;17(6):e0270003. doi: 10.1371/journal.pone.0270003 (PMC9202901; doi:10.1371/journal.pone.0270003)
Supplement: S2 File — (DOCX) [file pone.0270003.s003.docx]

**S2 File. Newcastle-Ottawa quality assessment scale used in the systematic review**

| Author | Year | Adequacy of case definition | Representativeness of the cases | Selection of Controls | Definition of Controls | Study controls for most important factor(s) | Study controls for second important factor(s) | Ascertainment of exposure | Same method of ascertainment for cases and controls | Non-Response rate | Overall quality result | | |
| --- | --- | --- | --- | --- | --- | --- | --- | --- | --- | --- | --- | --- | --- |
| Shin et al.^12^ | 2020 | ✱ | ✱ |  | ✱ | ✱ | ✱ | ✱ | ✱ |  | 7 | | |
| Glasauer et al. ^13^ | 2019 | ✱ |  |  | ✱ | ✱ | ✱ | ✱ | ✱ |  | 6 | | |
| Stosic et al.^14^ | 2019 | ✱ | ✱ |  | ✱ | ✱ | ✱ | ✱ | ✱ |  | 7 | | |
| Gaborit et al.^15^ | 2018 | ✱ | ✱ |  | ✱ | ✱ | ✱ | ✱ | ✱ |  | 7 | | |
| Gao et al. ^16^ | 2016 | ✱ | ✱ | ✱ | ✱ | ✱ | ✱ | ✱ | ✱ |  | 8 | | |
| Li et al. ^17^ | 2016 | ✱ | ✱ |  | ✱ | ✱ | ✱ | ✱ | ✱ |  | 7 | | |
| Yin et al. ^18^ | 2016 | ✱ |  |  | ✱ | ✱ | ✱ | ✱ | ✱ |  | 6 | | |
| Wang et al. ^19^ | 2016 | ✱ |  |  | ✱ | ✱ | ✱ | ✱ | ✱ |  | 6 | | |
| Chuchottaworn et al. ^20^ | 2015 | ✱ | ✱ | ✱ | ✱ | ✱ | ✱ | ✱ | ✱ |  | 8 | | |
| Elmi et al. ^21^ | 2015 | ✱ | ✱ | ✱ | ✱ | ✱ | ✱ | ✱ | ✱ |  | 8 | | |
| Mor et al. ^22^ | 2014 | ✱ | ✱ | ✱ | ✱ | ✱ | ✱ | ✱ | ✱ |  | 8 | | |
| Li et al. ^23^ | 2014 | ✱ | ✱ |  | ✱ | ✱ | ✱ | ✱ | ✱ |  | 7 | | |
| Zhao et al.^24^ | 2012 | ✱ | ✱ |  | ✱ | ✱ | ✱ | ✱ | ✱ |  | 7 | | |
| Coelho et al. ^25^ | 2012 | ✱ |  |  | ✱ | ✱ | ✱ | ✱ | ✱ |  | 6 | | |
| Ayaz et al.^26^ | 2012 | ✱ | ✱ |  | ✱ | ✱ | ✱ | ✱ | ✱ |  | | 7 |  |
| He et al. ^27^ | 2011 | ✱ | ✱ |  | ✱ | ✱ | ✱ | ✱ | ✱ |  | 7 | | |
| Fox et al. ^28^ | 2011 | ✱ | ✱ | ✱ | ✱ | ✱ | ✱ | ✱ | ✱ |  | 8 | | |
| Massi et al. ^29^ | 2011 | ✱ |  |  | ✱ | ✱ | ✱ | ✱ | ✱ |  | 6 | | |
| Balaji et al. ^30^ | 2010 | ✱ | ✱ |  |  | ✱ | ✱ | ✱ | ✱ |  | 6 | | |
| Diande et al. ^31^ | 2009 | ✱ |  | ✱ | ✱ | ✱ | ✱ | ✱ | ✱ |  | 7 | | |
| Shen et al. ^32^ | 2009 | ✱ | ✱ | ✱ | ✱ | ✱ | ✱ | ✱ | ✱ |  | 8 | | |
| O’Riordan et al. ^33^ | 2008 | ✱ | ✱ |  | ✱ | ✱ | ✱ | ✱ | ✱ |  | 7 | | |
| Tanrikulu et al. ^34^ | 2008 | ✱ |  |  | ✱ | ✱ | ✱ | ✱ | ✱ |  | 6 | | |
| De Souza et al. ^35^ | 2006 | ✱ | ✱ | ✱ | ✱ | ✱ | ✱ | ✱ | ✱ |  | 8 | | |
| El Sahly et al.^36^ | 2006 | ✱ | ✱ | ✱ | ✱ | ✱ | ✱ | ✱ | ✱ |  | 8 | | |
| Conaty et al. ^37^ | 2004 | ✱ | ✱ |  | ✱ | ✱ | ✱ | ✱ | ✱ |  | 7 | | |
| Schaberg et al. ^38^ | 1995 | ✱ | ✱ |  | ✱ | ✱ | ✱ | ✱ | ✱ |  | 7 | | |
| Pearson et al. ^39^ | 1992 | ✱ | ✱ |  | ✱ | ✱ | ✱ | ✱ | ✱ |  | 7 | | |
